# P-1745. Neuroparacoccidioidomycosis: A 12-Year Case Series of Paracoccidioidomycosis with Central Nervous System Involvement in an Endemic Region

**DOI:** 10.1093/ofid/ofaf695.1916

**Published:** 2026-01-11

**Authors:** Pedro A Villalba apestegui, Gustavo-Adolfo Méndez, Carla Niveyro, lucila maria COMPAÑY KEC, Cynthia Tomasino, Ricardo de Jesus Solari Maidana

**Affiliations:** Infectious Diseases Department, Hospital Escuela de Agudos Dr. Ramón Madariaga. Posadas, Misiones –Argentina, posadas, Misiones, Argentina; Hospital Escuela de Agudos Dr Ramón Madariaga, Posadas, Misiones, Argentina, Posadas, Misiones, Argentina; Madariaga Hospital, Posadas, Misiones, Argentina; Infectious Diseases Department, Hospital Escuela de Agudos Dr. Ramón Madariaga. Posadas, Misiones –Argentina, posadas, Misiones, Argentina; Infectious Diseases Department, Hospital Escuela de Agudos Dr. Ramón Madariaga. Posadas, Misiones –Argentina, posadas, Misiones, Argentina; Infectious Diseases Department, Hospital Escuela de Agudos Dr. Ramón Madariaga. Posadas, Misiones –Argentina, posadas, Misiones, Argentina

## Abstract

**Background:**

Paracoccidioidomycosis (PCM) remains a leading systemic mycosis in South America, disproportionately impacting rural and underserved communities. Central nervous system involvement—neuroparacoccidioidomycosis (NPCM)—is often underrecognized, delaying diagnosis and treatment. By sharing our 12-year institutional experience, we aim to raise clinical awareness, foster interdisciplinary collaboration and improve patient outcomes.

**Figure:**
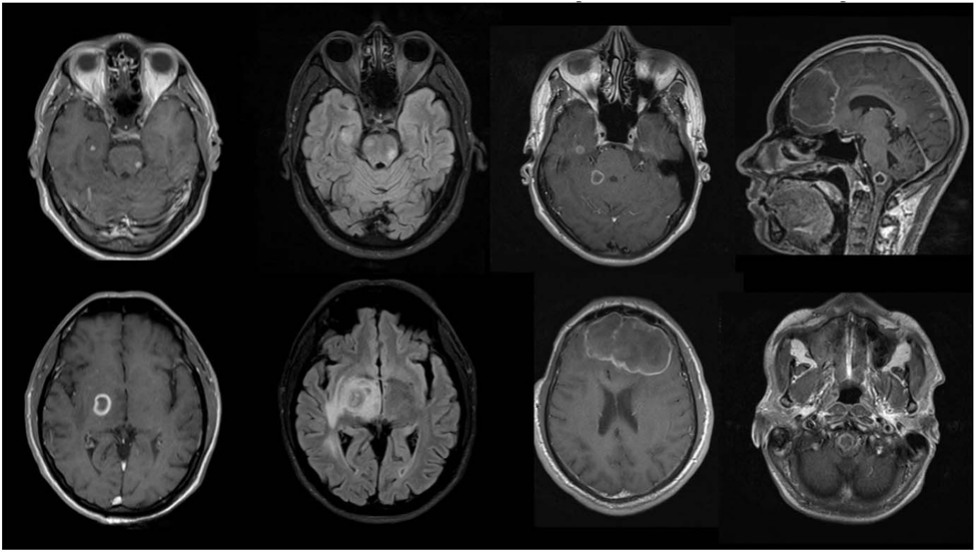
Contrast-Enhanced Brain MRI in Neuroparacoccidioidomycosis Axial and sagittal post-contrast T1-weighted MRI demonstrates round, peripheral ring-enhancing lesions with central low signal (A–C) and corresponding FLAIR hyperintensity indicating vasogenic edema (D–F). Note solitary lesions in the basal ganglia (A, D) and multiple lesions in the frontal lobe (B, E), all showing restricted diffusion on DWI (not shown). These imaging hallmarks should prompt consideration of NPCM in endemic settings.

**Figure:**
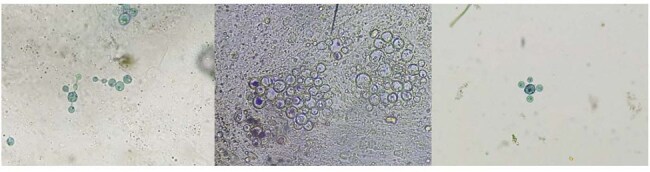
Microscopic Features of Paracoccidioides brasiliensis Direct KOH preparation (left) and culture smear (right) at 40× magnification reveal the classical “pilot-wheel” morphology: large, spherical yeast cells (8–20 µm) with multiple narrow-based buds arranged circumferentially.

**Methods:**

We performed a combined retrospective–prospective review of all PCM cases managed at Hospital Escuela de Agudos Dr. Ramón Madariaga from 2012 to 2024. NPCM was confirmed by cerebral biopsy (histopathology and/or culture) or by a consistent clinical–radiological profile. All patients underwent contrast-enhanced brain MRI, which was re-evaluated by specialist neuroradiologists. Recorded variables included lesion number, distribution, enhancement pattern, and diffusion characteristics.

**Results:**

Among 86 PCM patients, 13 (15.1%) developed NPCM (mean age 50.7 years, range 40–67; 100% male; 100% smokers; 75% regular alcohol use; 15.4% with significant comorbidities). Seventy-five percent presented neurological symptoms at diagnosis—most commonly headache with focal weakness (41.7%), paresthesias or gait disturbances (25%), and dizziness (25%). MRI showed solitary lesions in 53.8%, contrast enhancement in 92.3% (predominantly peripheral ring in 69.2%), and restricted diffusion in 83.3%. Diagnosis was confirmed by histopathology in 69.2% and by culture in 66.7%, with one case diagnosed by serology alone. Two patients died during hospitalization; all survivors began antifungal therapy, though long-term follow-up was limited.

**Conclusion:**

Over 12 years, approximately one in six PCM patients in this endemic region developed CNS involvement. Given the nonspecific presentation, a high index of suspicion—especially in individuals from endemic areas—is essential. Prompt contrast MRI with diffusion sequences and clear communication between infectious disease specialists and neuroradiologists can expedite diagnosis, guide targeted antifungal therapy, and improve prognosis. We hope this series inspires earlier recognition and collaborative care strategies to reduce morbidity and mortality associated with NPCM.

**Disclosures:**

All Authors: No reported disclosures

